# Quantitative PCR for parasitemia monitoring and preemptive therapy in patients with Chagas disease and autoimmune rheumatic disorders undergoing biologic treatment: a case series

**DOI:** 10.1590/0037-8682-0419-2024

**Published:** 2025-06-02

**Authors:** Noemia Barbosa Carvalho, Vera Lucia Teixeira de Freitas, Nadia Emi Aikawa, Lucas Teixeira Vieira, Rita Cristina Bezerra, Adriana Coraccini Tonácio de Proença, Karina Bonfiglioli, Érika Yoshie Shimoda Nakanishi, Hermes Ryoiti Higashino, Maria Aparecida Shikanai-Yasuda

**Affiliations:** 1 Universidade de São Paulo, Divisão de Moléstias Infecciosas do Hospital das Clinicas da Faculdade de Medicina, São Paulo, SP, Brasil.; 2 Universidade de São Paulo, Departamento de Infectologia e Medicina Tropical da Faculdade de Medicina, São Paulo, SP, Brasil.; 3 Universidade de São Paulo, Laboratório de Investigação Medica em Imunologia (LIM 48) do Hospital das Clinicas da Faculdade de Medicina, São Paulo, SP, BR.; 4 Universidade de São Paulo, Divisão de Reumatologia, Hospital das Clinicas da Faculdade de Medicina, São Paulo, SP, Brasil.; 5 Universidade de São Paulo, Laboratório de Investigação Medica em Parasitologia (LIM 46), Hospital das Clinicas da Faculdade de Medicina, São Paulo, SP, Brasil. .

**Keywords:** Chagas disease, Autoimmune rheumatic diseases, T. cruzi, Parasitemia, qPCR

## Abstract

Patients with chronic Chagas disease (CD) and autoimmune rheumatic disorders (ARD) receiving immunosuppressive therapy are at risk for CD reactivation (CDR). This study monitored parasitemia over 9-121 months in six patients with CD and ARD using conventional and quantitative PCR and parasitology. Five patients showed parasitemia; two had elevated levels of parEq/mL (49-458.7). One patient received benznidazole with a marked decrease in parasitemia; in the other, treatment was discontinued after 12 days due to toxicity. No CDR occurred. These findings support the need for qPCR standardization for preemptive therapy and suggest that benznidazole may prevent CDR in patients with high parasitemia.

## INTRODUCTION

Chagas disease (CD) is increasingly prevalent in urban centers across continents due to migration of chronically infected individuals to large cities in endemic and non-endemic regions^1^ ^,^ ^2^. Autoimmune diseases have been identified in 0.8-1.3% of the 27% of immigrants affected by CD in Geneva, Switzerland^1^ ^,^ ^2^. 

Patients with chronic CD, typically presenting with low but persistent parasitemia^3^, are at risk for CD reactivation (CDR) when associated with autoimmune rheumatic disorders (ARDs), AIDS, hematologic diseases, neoplasms, or organ and tissue transplantation. CDR is a potentially life-threatening complication with high mortality^3^. 

A refined understanding of ARD pathophysiology has led to the development of novel disease-modifying antirheumatic drugs (DMARDs), including biologics and targeted synthetic DMARDs^4^. These drugs interfere, to varying degrees, with T lymphocytes and macrophage functions, which are central to controlling parasite multiplication, thereby increasing parasitemia and the risk of reactivation. 

First-line therapy for ARD includes glucocorticoids and conventional synthetic immunosuppressants, such as methotrexate, leflunomide, cyclosporine, azathioprine, mycophenolate mofetil, and cyclophosphamide. In patients with inadequate remission or low disease activity, advanced therapies are indicated, including targeted synthetic DMARDs, such as biologic agents (tumor necrosis factor-alpha [TNFα] inhibitors [adalimumab, certolizumab, etanercept, golimumab, and infliximab]), T-cell inhibitors (abatacept), B-lymphocyte inhibitors (rituximab), and agents with alternative mechanisms of action beyond TNFα inhibition, such as anti-CTLA4, anti-IL6, anti-IL17, anti-IL12/23, anti-CD20, and anti-Blys5-7^4^.

A review of the literature on CD and ARD highlights several issues: (1) Ten reported cases of CDR in patients with ARD, including systemic lupus erythematosus, rheumatoid arthritis (RA), psoriatic arthritis, systemic vasculitis, and limited cutaneous sclerosis^5^ ^-^ ^9^; (2) Eight additional patients with elevated parasitemia^10^ ^-^ ^11^, suspected of having CDR; (3) Seventy-eight patients without CDR^1^ ^,^ ^2^ ^,^ ^7^ ^,^ ^11^ ^-^ ^13^; (4) The lack of quantitative PCR (qPCR) standardization in non-immunosuppressed and immunosuppressed patients, limiting cross-study comparisons^10^ ^,^ ^11^ ^,^ ^13^. 

Given the potentially increased risk of CDR, this study aimed to assess qPCR as a marker for preemptive benznidazole therapy in six patients with CD and ARDs receiving biologic treatment.

## CASE REPORTS

This prospective observational study included six consecutive patients with ARD and concomitant CD, followed between 2009 and 2023 at the Infectology and Rheumatology Clinics of a university hospital. Diagnosis of *T. cruzi* infection was confirmed using two different serological tests (ELISA, indirect immunofluorescence, hemagglutination, or chemiluminescence)^14^.

Parasitemia was monitored using molecular and indirect parasitological methods (blood culture, xenodiagnosis, or both), and direct microscopic examination of blood^3^. Conventional PCR (cPCR) amplified a 330 bp minicircle sequence using S35 and S36 primers with modifications^14^. qPCR was performed with TCZ3 and TCZ4 primers to amplify a 149 bp genomic fragment using SYBR Green-based qPCR^14^. The positivity rate of indirect parasitological methods was 19.4% in patients with chronic CD and 57.5% in HIV-coinfected patients without CDR; molecular methods showed 37.8% and 70.2% positivity, respectively, with 100% specificity^14^. In prior qPCR studies^14^, baseline levels in chronic cases reached up to 11.0 parasite Equivalents/mL (parEq/mL), whereas in coinfected patients without CDR, stable parasitemia reached up to 67.0 parEq/mL, and variable parasitemia reached up to 390 parEq/mL^14^. 

Benznidazole (5-7 mg/kg/day for 60 days)^14^ was used as preemptive antiparasitic therapy in cases with increased parasitemia, defined as parEq/mL levels higher than the maximum observed in immunosuppressed patients without CDR (> 67 parEq/mL)^14^ by qPCR, and a relative increase compared to the patient's previous baseline control.

Inclusion criteria: (1) Confirmed diagnosis of CD by two serological tests and an autoimmune disease diagnosed by gold standard methods; (2) Evaluation of parasitemia in at least three samples using molecular methods. Exclusion criteria: Patients followed for < 6 months or those who did not undergo the recommended tests to characterize the clinical form of CD.

This study was approved by the Ethics and Research Committee of our institution (protocol numbers 1043/07 [12/05/2007] and 1298/06 [01/17/2007]). All patients provided written informed consent. Data were collected anonymously from medical records.

Six patients with ARD and CD were followed up for 9-121 months (median, 85.5 months [interquartile range 25%-75%, 64.25- 110.5]) (Table 1). 

**TABLE 1:** Clinical profile, immunosuppressive therapy, and *T. cruzi* parasitemia monitoring by cPCR, qPCR, and parasitological methods in six patients with autoimmune rheumatic disorders.

Case	Age Sex	Diagnosis, date (month/year) Clinical activity	Treatment (month/year)	Laboratory exams and disease activity	Chagas disease evaluation (clinical form)	cPCR/ Blood culture/Xeno-diagnosis X month/year	qPCR/D	*months follow-up/medicines BNZ
P1	76 F	Rheumatic polymyalgia 01/2009. Temporal arteritis?	PRE 10 mg/d (01-03 /2009) + ETA 50 mg/week (01/ 2009 to 01/2010)		(Cardiac + megaesophagus). Normal ECG, Holter: 2^nd^-degree AVB, intermittent Mobitz II.	05/2009 N/B+XN	U/DN	
					Echo-discrete diastolic dysfunction,	07/2009N/B+XN 10/2009 N/BNAXNA	U/NA U/NA	
			MTX until 07/2010		Esophagus-hypotonia-lower sphincter: 2012 and megaesophagus: 2016	02.2010 N/BNXN	U/DN	9*
P2	54 F	Antisynthetase-like syndrome (polymyositis 05/2010 - Raynaud phenomenon 03/2011: poly-neuropathy, pneumopathy 05/11 - Dysphagia, muscle weakness, myalgia, myopathy (biopsy) 12/2011: microangiopathy	MTX - 03/2011 to 04/2012 10-25 mg/week MP - 3 g (04/2011), AZA 100 mg/d, followed by PRE 40-80 mg/d (2011), 10-60 mg (2012), suspended (03/2014)	Anti-ku antibody +, 02/11 ENM chronic myopathy, peripheral polyneuropathy CPK 6895 U/L, Aldolase 83/U/L. Anti-nuclear antibody +, CPK 1841 U/L	(Cardiac + megaesophagus) ECG - RBB - ASRB 2011-2017 ECG - RBB - ASRB 2011-2017	05/2011 +/BNAXN 07/2011+/BNXN 11/2011 +/BNXN	65.4/DN 17.5/DN 2.84/DN	24*MPRED PRED 40 mg/d
		04/2012: worsening muscle weakness	RIT (07/2013) (2^nd^ cycle), PRED 20 mg/d, MTX 20 mg/d	Negatives ENA, Scl70, DNA, Sm, Ro	Holter 2014 - AVB 2^nd^ degree, RVV +ASBB 09/2014	03/2012 +/BNAXNA 05/2012 +/B+XN	7.55/DN 10.6/DN	
			RIT (01/2014), PRED suspended (03/2014)	Negatives La, ACL, LAC, Jo1 (anti-histidil tRNA sintetase)	Echocardiogram - LV hypertrophy - 2011-2012	08/2012 +/B+XN 01/2013 +/ BNXN 03/2013 +/BNX+	6.2/DN 27.5/DN 40.3/DN	22*/PRED 20-25 mg/d
			Failure 3 drugs, introduced RIT (07/2012) (4 cycles) - 01/2014 RIT, PRE 25 mg/d - 01/2013		Echocardiogram 2014 - akinesia (basal segment - inferior septum)	09/2013 +/B+XNA 01/2014 +/B+XN	1.65/DN 6.15/DN	
					Esophageal manometry - hypotonia, motor activity absent	09/2013 +/B+XNA 01/2014 +/B+XN	1.65/DN 6.15/DN	
					In middle, distal segments - 2014	02/2015 +/BNAXNA	U/DN	
			MTX 25 mg/week (03/2015 - 09/2016)	Improvement	Esophagoscopy - Grade I	10/2015 +/BNAXNA	25.3/DN	
		Pain and lymphnodomegaly			Megaesophagus 2016	05/2016 +/BNAXNA	76.4/DN	60*/PRED 40 mg/d
		- reactional lymphadenitis		10.2016-Leucocytes 2740, platelets 146000/mm^3^,	10/2016 -12 days, BNZ 300 mg/d - suspended due to fever, rash,	11/2016 N/BNAXNA	NA/DN	65* BNZ 12 days
			MTX 10-30 mg/week - 2017 to 01/2019	Hb 13.2 g/dL	13 days leukopenia	02/2017 +/BNAXNA	2.19/DN	
			MTX 20 mg/week - 01/2018			01/2018 +/BNAXNA	46.3/DN	80*MTX
		enlarged mediastinal, axillary lymph nodes,	MTX - 10 mg/week. No PRED since 07/2016	05/2018: Anti-synthetase syndrome (anti-ku+)		05/2018 +/BNAXNA	63.9/DN	84 *MTX
		interstitial pneumopathy				06/2018 +/BNAXNA	7.7/DN	
		Pain and joint heat (04/2019), stable pneumopathy	MTX increased 20 mg/day (04/2019)			04/2019 +/BNAXNA	46.95/DN	93 *MTX
		Arthralgia and joint edema (03/2021)	MTX 20 mg/day until 01/2021 MTX - PRE 03/2021			12/2019 +/BNAXNA 06.2021+/BNAXNA	23.04/DN 11.12 DN	121*
P3	57 F	Rheumatoid arthritis (2008) - Ac antiCCP +	LEF 20 mg/d - 09/2013-10/2017		(Megaesophagus) 2013 - ECG and echocardiogram - normal	01/2014 N/BNXN	U/DNA	
			MTX 10-15 mg/week - 2011 -11/2013		2016 - Reduced peristalsis, discrete reflux, and nonspecific	03/2014 N/BNXN	U/DNA	
			HQ 400 mg/d - 12/2013- 05/2019		dysmotility in XR of stomach, esophagus, and duodenum	07/2014 N/BNXNA	U/DN	
			MTX 03/2015		Esophagogram: increased	01/1015 N/BNAXNA	U/DN	
			PRE 10 mg/day - 01/2016		esophageal caliber and slow	02/2016 N/BNAXNA	U/DNA	
			suspension gastric intolerance		empty time	05/2017 +/BNAXNA	U/DN	40*PRED 10 mg/d
						03/2018 N/BNAXNA	U/DNA	
						05/2019 N/BNAXNA	U/DN	64*
P4	67 F	Primary Sjogren syndrome (2010)	MTX 17.5-25 mg/week: 09/2009-02/2014, 10 mg/week until 2023	Anti-nuclear antibody +	(Non-cardiac form)	01/2014 N/BNAXNA	U/DNA	
		polyarthritis, rheumatoid vasculitis	PRE 2.5-10 mg/day: 2009-2015, HQ 400 mg/d (02/14) MTX 10 mg/week	anti-SSA (Ro) antibody	2014 - Echocardiogram early diastolic dysfunction*	03/2014 N/BNXN	U/DNA	
		interstitial pneumopathy	HQ: 06/2010-02/2015	anti-SSP (La) antibody	2014, 2016,2017 - Normal ECGs	05/2014 N/BNXN	0.47/DNA	3*PRED 10mg/d
		neuropathy	COL 0.5 mg/d: 03/2014-02/2015	Rheumatoid factor - Negative	2016 - No megaesophagus	10/2014 N/BNXN	U/DN	
		xerostomia	PRE was reintroduced in 04/2016	CCP - Negative		01/2016 N/BNXN	U/DNA	
		xerophthalmia	pneumopathy			09/2016 N/BNXN	U/DN	
		xerotrachea				05/2017 N/BNXN	NA/DN	
						07/2018 N/BNXN	U/DN	
						03/2019 N/BNXN	NA/DN	
			05/14-2023: HQ 400 mg +			10/2020 N/BNXN	NA/DN	
			PRE 7.5 mg/d			11/2022 N/BNXN	U/DNA	106*
P5	62 F	Rheumatoid arthritis (2005)	CSP 100 mg/d (10/2009-06/2013). MTX 25 mg/week (2005-2014)	Rheumatoid factor + DAS 28: 7.88 - 06/2013	(Cardiac form)	01/2014 N /BNXNA	U/DNA	
		Rheumatoid vasculitis	LEF-neuropathy (2009) ABA (12/2013-03/2014)	SDAI 5, CDAI 5, AVG 50 DAS 28: 6.1 - 12/2013	2015, 2019 - Frequent atrial extrasystole ECG.	10/2014 N /BNAXNA	U/DN	
		Treated pulmonary tuberculosis	PRE 5-20 mg/day (10/2009 - 11/2014)		Echocardiogram discrete alteration of left ventricular	10/2015 N /BNAXNA	U/DN	
		Mild obstructive pulmonary disease, diabetes mellitus (II)	TOC (08/2014-08/2018). (10/2014) - TOC, ME 25 mg/week, PRE 5 mg/day		relaxation. 2020 - Holter- non-sustained atrial tachycardia and rare	05/2018 N /BNAXNA	NA/DNA	
		Heart failure (07/2019)	TOC (08/2014-08/2018). (2019) - TOC 4/4 weeks, PRE 10 mg/d, MTX 10 mg/d	DAS 28: 6.6 - 08/2014	episodes of aberrant intraventricular conduction.	06/2019 N/BNAXNA	NA/DNA	65*
P6 M	43 F	Rheumatoid arthritis (2000)	MTX, HQ, RIT before 2010 -2011, TOC (2011)	Positive rheumatoid factor C-reactive protein: 46 mg/L	(Cardiac form) 2014- RBB, junctional escape, low voltage, changeable	04/2014 +/BNAXNA	409,5/DN	AZA, PRE 10 mg/d
		Desquamative pneumonitis	PRE 10 mg/d (2012) - 15 mg/day (10/2017)	DAS 28: 6.05 (10/2012)	Atrial pacemaker - ECG	09/2014 +/B+XNA	29.5/DN	
		12/2020 - Pulmonary biopsy	LEF 2012-06/2013 (neuropathy)	DAS 28: 4.79 (07/2014)	2014 - Rare atrial and ventricular	10/2014 +/B+XNA	6.3/DN	
		Chronic non-specifc interstitial pneumonia	AZA 100-150 mg/d (12/2012 -09/2016)	DAS 28: 5.43 (04/2015); 7.05 (06/2015); 3.8 (10/2015)	Extrasystoles, and supraventricular tachycardias	03/2015 +/BNAXNA	51.0/DN	*11/AZA, ABA, PRE 10 mg/d
		Progressive worsening of interstitial pneumonia	ABA 500 mg (09/2014-10/ 2015)	DAS 28: 6.4 (11/2015)	2014 - Left ventricular ejection fraction 60% - Echocardiogram Esophagoscopy - Normal	10/2015 +/BNAXNA	223.0/DN	18*/AZA,ABA, PRE 10 mg/d
		CT scans and digital clubbing,	TCO (05/2015-02/2016) - Falha	DAS 28: 6.4 (02/2016)	2013-2017 - Chest Xray - interstitial pneumopathy associated with ARD	02/2016 +/BNAXNA	458.7/DN	22*/AZA.TOC, PRE 10 mg/d
		interstitial pneumopathy	TOF (10/2015-02/2016)			04/2016 N/BNAXNA	U/DN	24*/BNZ 03.2016 30d
			AZA, PRE (09/2016)			07/2016 +/BNAXNA	U/DN	
			RIT (11/2017)			09/2016 N/BNAXNA	U/DNA	
			PRE 15 mg (02/2018)	DAS: 3.8 (02/2018)		05/2017 +BNAXNA	U/DN	
			MP 100 mg (02/2018)	PCR 46		01/2018 +/BNAXNA	U/DN	45*/MP
			RIT (11/2018 and 08/2022-09/2023)			06/2018 +/BNAXNA	0.08/DN	
			TOC (03/2019-05/2022)			12/2018 +/BNAXNA	0.40/DN	
			TOC (03/2020-02/2022)	hepatotoxicity		03/2019 +/BNAXNA	U/DN	
			PRE 10-20 mg/d (08/2020-05/2022) - ?40 mg /day+MPRED	Severe evolution pneumopathy, arthralgia,		12/2020 +/BNAXNA	0.12/DN	80*MP+ PRE 40 mg/d
			PRED 40 mg/d (08/2022) dyspnea	lung transplant no		09/2022 +/BNAXNA	3.1/DN	
		Lung amyloidosis	MP 100 mg (08/2022) dyspnea: MMF (12/2022) toxicity	Indicated: renal insufficiency		12/2022 +/BNAXNA	U/DN	104*MP
		Renal amyloidosis	PRE low doses (09/2023)			08/2023 +/BNAXNA	2.3/DN	112*

**F:** female; **M:** male; **(y):** years; **cPCR:** conventional polymerase chain reaction (qualitative); **qPCR:** quantitative PCR; **U:** undetectable; **D:** direct parasitology; *months of follow-up; **PRE:** prednisone; **MP:** methylprednisolone; **MTX:** methotrexate; **ETA:** etanercept; **AZA:** azathioprine; **CSP:** cyclosporine; **COL:** colchicine; **RIT:** rituximab; **HQ:** hydroxychloroquine; **LEF:** leflunomide; **ABA:** abatacept; **TOC:** tocilizumab; **TOF:** tofacitinib; **N:** negative; **ECG:** electrocardiogram; **Echo:** echocardiogram; **B:** blood culture; **NA:** not available; **X:** xenodiagnosis; **D:** direct microscopy; **BNZ:** benznidazole; **ku:** Ku70/Ku80; **DNA:** binding protein; **CPK:** creatine phosphokinase; **CRP:** C-reactive protein; **RBB:** right branch block; **ASBB:** anterosuperior branch block; **AVB:** atrioventricular block; **CCP:** cytric citrullinated peptide; **CPK:** creatine phosphokinase; **anti ENA:** nuclear extrated antibodies (RNP, Sm, SSa/Ro, SSb/L); **anti Ro (SSa) and La (SSb):** anti RNA antibodies; Scl70-anti-toporoisomerase 1, **antiI-SSm:** anti-Smith antibody; **aCL:** anticardiolipin; **LAC:** lupus anticoagulant; **anti JO:** anti-histidine tRNA synthetase; **SDAI:** simple disease activity index; **CDAI:** clinical disease activity index; **DAS:** disease activity score.

All patients were female, aged 43-76 years; two had cardiopathy, two had cardiopathy with megaesophagus, one had megaesophagus, and one had a non-cardiac form of CD^3^ (Table 1). 

Five patients had *T. cruzi* parasitemia confirmed by parasitological or molecular method^14^, and two showed elevated parasitemia by qPCR (76.4 and 458.7 parEq/mL). The first patient (P2) with severe polymyositis and antisynthetase syndrome received methylprednisolone followed by prednisone (>20 mg/day) during 2/5 relapses, combined with methotrexate, or rituximab and methotrexate alone in three relapses. The second patient (P6) with RA received prednisone (10-15 mg/day) and azathioprine (100 mg/day) in four relapses, combined with azathioprine in one relapse, abatacept in two, and tocilizumab in one. 

### Patient 1

A 76-year-old female patient was diagnosed with rheumatic polymyalgia and temporal arteritis and treated with prednisone, methotrexate, and etanercept without clinical improvement. CD progressed to cardiopathy (2011) and megaesophagus (2016). Parasitemia remained negative by direct microscopy, cPCR, qPCR, blood culture, and xenodiagnosis for 9 months, except for two positive blood cultures in 2009.

### Patient 2

A 54-year-old female was diagnosed with polymyositis and antisynthetase syndrome associated with pneumopathy. She was prescribed methotrexate, methylprednisolone, azathioprine, prednisone (up to 60 mg/day), cyclosporine, and rituximab. 

CD had progressed to cardiopathy and megaesophagus*. T. cruzi* parasitemia remained positive for 10 years.

Antiparasitic treatment was considered due to increasing parasitemia despite severe myopathy. With an increase to 76.4 parEq/mL from basal levels of 1.65-7.7 parEq/mL, benznidazole (5 mg/kg/day) was prescribed on October 2016 but discontinued after 12 days due to fever and generalized erythema; leukopenia was observed the day after discontinuation. Parasitemia was monitored thereafter, with transient elevations up to 63.9 parEq/mL in the absence of an effective alternative antiparasitic drug with no toxic effects.

### Patient 3

A 57-year-old female was diagnosed with RA in 2008 and treated with prednisone, leflunomide, methotrexate, and hydroxychloroquine. She had Chagasic megaesophagus. *T. cruzi* parasitemia was monitored for 64 months, with one of eight cPCR results testing positive.

### Patient 4

A 67-year-old female was diagnosed with Sjögren’s syndrome, polyarthritis, rheumatoid vasculitis, interstitial pneumopathy, and bilateral keratitis. She was prescribed methotrexate, prednisone, and hydroxychloroquine. CD was classified as non-cardiac. *T. cruzi* parasitemia was monitored for 106 months, with only 1/7 qPCRs showing a low par Eq/mL level (Table 1).

### Patient 5

A 62-year-old female with RA (2005) was treated with methotrexate, cyclosporine, abatacept, prednisone, tocilizumab, and hydroxychloroquine. A cardiac form of CD was diagnosed, and parasitemia remained negative for 65 months. 

### Patient 6

A 43-year-old female was diagnosed with RA associated with desquamative pneumonitis. She had previously received methotrexate and hydroxychloroquine, followed by rituximab, and was later switched to tocilizumab. The patient was subsequently prescribed leflunomide, azathioprine, abatacept, and tofacitinib. 

Cardiac CD was confirmed. Parasitemia monitoring revealed transient peaks between April 2014 and October 2015. In February 2016, 458.7 parEq/mL was detected, and benznidazole (300 mg/day) was prescribed for 60 days. Parasitemia decreased to undetectable or very low qPCR levels, with no recurrence of high parasitemia, despite high-dose methylprednisolone use during the 84-month follow-up post-therapy.

## DISCUSSION

This study found positive parasitemia in 83.3% of patients (5/6), which was higher than that observed in chronic non-immunosuppressed cases^14^ and comparable to that in *T. cruzi*-HIV coinfected patients^14^. This frequency also exceeded the 53.8% reported in one case series^11^, while such data were not available in other series^1^ ^,^ ^2^ ^,^ ^7^ ^,^ ^11^.

No cases of CDR were identified according to standard parasitological or quantitative molecular methods, contrasting with the 11.8% (6/51) reported previously^7^ (Table 1). In most cases, corticosteroids > 20 mg/day were not prescribed for extended periods as first-line therapy, as they interfere with cellular and humoral immune response^4^. 

In two patients, parasitemia levels of 65.4 parEq/mL (P2) and 409.5 parEq/mL (P6) at the beginning of follow-up were transient and decreased to 2.84 and 6.3 parEq/mL, respectively, without antiparasitic treatment (Table 1). No clinical signs, symptoms, or positive direct parasitological methods indicative of CDR were observed. 

In ARD, comparing high parasitemia levels across studies is challenging due to the lack of qPCR standardization. Parasitemia levels of 57.4^13^ and > 50 parEq/mL^11^ have been reported as markers of CDR, whereas levels around 1,000 copies were considered low and not indicative of CDR^10^. In contrast, high values ranging from 996 to 5,575 parEq/mL^11^ may suggest CDR.

Standardization of qPCR as a marker for CDR has been infrequently reported across different laboratories in patients with CD with varying levels of immunosuppression (chronic CD and immunosuppressed CD patients with or without CDR)^14^. In patients with chronic CD, the maximum parasitemia detected was 11.0 mEq/mL. Among CD-HIV coinfected patients without CDR, parasitemia reached up to 67.0 mEq/mL in those with stable levels, and 390 parEq/mL in a group with variable parasitemia^14^. 

Notably, preemptive antiparasitic therapy in P6 was followed by a marked decrease in parasitemia to undetectable or 2.3 parEq/mL during the long follow-up period, as reported in patients with high parasitemia (other ARD patients with CD^11^, *T. cruzi* HIV coinfection^14^, stem cell transplantation, and medullary aplasia without CDR^15^ ^-^ ^16^). None of these cases developed CDR. However, the toxicity of antiparasitic drugs must be considered, particularly in patients with systemic myopathy (P2). No similar long follow-up data after treatment have been reported.

In this context, based on prior studies^13^ ^-^ ^16^ and the present series, absolute qPCR levels ≥ 70 -100 parEq/mL or increased parasite levels relative to a patient's baseline may indicate the need for preemptive therapy, warranting confirmation in larger cohorts of patients with of ARD and CD.

## CONCLUSION

This study confirmed the utility of qPCR as a marker of increased parasitemia. A marked and sustained reduction in qPCR levels was observed following benznidazole therapy, despite subsequent high-dose corticosteroid use. Unlike previous reports, no cases of CDR occurred, possibly due to the less frequent use of high-dose corticosteroids for long periods.
